# Association between clustering of cardiovascular risk factors and left ventricular geometric remodeling in Chinese children

**DOI:** 10.3389/fcvm.2023.1236730

**Published:** 2023-08-17

**Authors:** Qin Liu, Huan Wang, Min Zhao, Cheng Zhang, Pascal Bovet, Bo Xi

**Affiliations:** ^1^ Department of Ultrasound, Children’s Hospital of the Capital Institute of Pediatrics, Beijing, China; ^2^ Institute of Child and Adolescent Health, School of Public Health, Peking University, Beijing, China; ^3^ Department of Toxicology and Nutrition, School of Public Health, Cheeloo College of Medicine, Shandong University, Jinan, China; ^4^ Key Laboratory of Cardiovascular Remodeling and Function Research, Chinese Ministry of Education, Chinese National Health Commission and Chinese Academy of Medical Sciences, The State and Shandong Province Joint Key Laboratory of Translational Cardiovascular Medicine, Department of Cardiology, Qilu Hospital, Cheeloo College of Medicine, Shandong University, Jinan, China; ^5^ Center for Primary Care and Public Health (Unisanté), University of Lausanne, Lausanne, Switzerland; ^6^ Department of Epidemiology, School of Public Health, Cheeloo College of Medicine, Shandong University, Jinan, China

**Keywords:** cardiovascular risk factor, children, geometric remodeling, left ventricle, Chinese

## Abstract

**Background:**

Several cardiovascular (CV) risk factors are reported to be associated with abnormal cardiac structure in children and adults. However, no study has assessed the association between clustering of multiple CV risk factors and left ventricular geometric (LVG) remodeling. We examined the association between clustering of CV risk factors and LVG remodeling among Chinese children.

**Methods:**

This cross-sectional study included 1,406 children aged 6–11 years. Clustering of CV risk factors was quantified as the sum of the number of five CV risk factors (abdominal obesity, elevated blood pressure, high fasting blood glucose, high triglycerides and low high-density lipoprotein cholesterol). Based on left ventricular mass index and relative wall thickness (RWT), left ventricular hypertrophy (LVH), high RWT and LVG remodeling [concentric remodeling (CR), eccentric hypertrophy (EH) and concentric hypertrophy (CH)] were defined.

**Results:**

Compared to participants without CV risk factor, those with 1, 2 and ≥3 risk factors were at increased risk of LVH [ORs (95% CIs): 3.49 (2.19–5.56), 5.53 (3.20–9.55), and 19.19 (9.67–38.08), respectively]; corresponding values for high RWT were 2.47 (1.63–3.74), 3.76 (2.25–6.27), and 5.47 (2.65–11.28). Similar associations between clustering of CV risk factors and LVG remodeling were found [CR: 1.71 (1.06–2.76), 2.83 (1.54–5.18), and 3.82 (1.37–10.62); EH: 2.42 (1.42–4.11), 4.23 (2.24–7.96), and 16.86 (7.70–36.92); CH: 14.92 (4.41–50.47), 23.15 (6.32–84.83), and 71.19 (17.09–296.56)].

**Conclusion:**

CV risk factors in isolation and combination were associated with an increased risk of LVH, high RWT and LVG remodeling among children, emphasizing the need to consider multiple risk factors when assessing the risk of cardiac outcomes.

## 1. Introduction

Left ventricular hypertrophy (LVH) is a common target organ damage in youth with hypertension (1). LVH and left ventricular geometric (LVG) remodeling, which are surrogate markers of abnormal cardiac structure, and are independently associated with cardiovascular disease (CVD) morbidity and mortality (2, 3), stroke (4, 5) and cognitive impairment (6, 7).

Previous studies have documented the association between several cardiovascular (CV) risk factors separately and left ventricular structure in both children (8, 9) and adults (10, 11). Indeed, abdominal obesity (12, 13), elevated blood pressure (9, 14), triglycerides (TG) and high-density lipoprotein cholesterol (HDL-C) (15, 16) are independently associated with cardiac structural remodeling in youth. Importantly, these CV risk factors usually co-exist in the population (17, 18) and the clustering of several CV risk factors could have a larger effect on CVD than the sole presence of individual CV risk factor (19). Although the clustering of at least three CV risk factors is defined as metabolic syndrome (MetS), this definition remains controversial. MetS is a binary definition (yes: number of the CV risk factors ≥3 vs. no: number <3) while those with 1 or 2 CV risk factors cannot be simply recognized as metabolicaly healthy. In 2017, the American Academy of Pediatrics also emphasized the need to consider CV risk factors clustering rather than MetS itself (20). Furthermore, a recent review also recommended a validated and unified continuous CV risk score for children (21). Accordingly, a continuous risk score of the clustering of the same five components from MetS may better identify CVD risk. Indeed, an international study of 2,427 children and adolescents aged 6–17 years showed that clustering of CV risk factors performed better than MetS in identifying high carotid intima-media thickness (cIMT, a surrogate marker of subclinical atherosclerosis) (22).

In this study, we investigated the association of clustering of CV risk factors (including the five risk factors used in the definition of MetS) with LVH, high relative wall thickness (RWT), and LVG remodeling [including concentric remodeling (CR), eccentric hypertrophy (EH), and concentric hypertrophy (CH)] among Chinese children aged 6–11 years.

## 2. Materials and methods

### 2.1. Participants

Data came from the baseline survey of “Huantai Childhood Cardiovascular Health Cohort Study” conducted in Huantai County, Zibo City, Shandong Province, China, conducted from November 2017 to January 2018. The survey has been described elsewhere (23). In brief, 1,406 children aged 6–11 years from one primary school were included in this study. All children and their parents/guardians provided written informed consent before examinations. Collected data included demographic information (sex and age), anthropometric examinations [height, weight, waist circumference (WC) and blood pressure (BP)], lifestyle factors (sleep duration, screen time, physical activity and intake of vegetables and fruits), fasting blood assays (glucose and lipid profiles) and an echocardiogram examination. The study protocol was approved by the institutional review board at the School of Public Health, Shandong University (approval number: 20160308).

### 2.2. Collections of CV risk factors

Anthropometric examinations were performed by trained staff according to a standard protocol in the school setting. Height and weight were measured twice in light clothes without shoes using a calibrated scale with stadiometer (HGM-300; Shengyuan Co. Ltd., China). WC was measured twice at 1 cm distance above the horizontal level of umbilicus using a flexible plastic tape. Mean values of height, weight, and WC, as well as body mass index (BMI, kg/m^2^), respectively, were calculated for data analysis. The BP in a seated position was measured three times consecutively at the heart level at about 20-second intervals, using the validated and calibrated electronic sphygmomanometer (HEM 7012; Omron, Osaka, Japan) (24). The last two BP readings were averaged for data analysis.

Venous blood samples were drawn after an overnight fast (for at least 10 h). Fasting blood glucose (FBG), TG and HDL-C were assayed using an automatic analyzer (Beckman Coulter AU480; Mishima, Shizuoka, Japan).

### 2.3. Measurements of left ventricular structure indices

A same experienced sonographer who was blinded with the study protocol measured the left ventricular structure using a portable color Doppler ultrasound machine (CX30; Royal Philips, Amsterdam, the Netherlands) equipped with an S4-2 convex array transducer (frequency of 2–4 MHz), according to recommendations proposed by the American Society of Echocardiography (25). Using the R-wave apex of the electrocardiogram as the standard phase, the left ventricular end-diastolic internal dimension (LVID) was obtained by measuring the echogenicity of the left ventricular septum from the left ventricular surface to the left ventricular posterior wall. In addition, the vertical distance between the two points on the time axis was measured from the upper edge of the front edge echo line to the upper edge of the rear edge echo line of the measured structure to obtain interventricular septal thickness (IVST) and left ventricular posterior wall thickness (LVPWT). Intra-observer reproducibility of IVST values [intra-class correlation coefficient (ICC) = 0.92] and LVPWT values (ICC = 0.95) was evaluated in the same 20 subjects by the same sonographer who measured twice. Left ventricular mass (LVM, g) was calculated using the Devereux's formula as 0.8 × 1.04 × [(IVST + LVID + LVPWT)^3^−(LVID)^3^] + 0.6 (26). Additionally, left ventricular mass index (LVMI, g/m^2.7^) was calculated as LVM divided by height to the power of 2.7 (27). RWT was calculated as (LVPWT + IVST)/LVID (28).

### 2.4. Definitions of CV risk factors and LVG patterns

CV risk factors including abdominal obesity, elevated BP, high FBG, high TG and low HDL-C were defined separately as recommended by the Society of Pediatrics, Chinese Medical Association (29). Abdominal obesity was defined as WC ≥90th percentile values for sex and age (30, 31). Elevated BP was defined as systolic and/or diastolic BP ≥95th percentile values for sex, age and height (32). High FBG (≥5.60 mmol/L), high TG (≥1.47 mmol/L) and low HDL-C (<1.03 mmol/L) were defined according to recommendations from the Society of Pediatrics, Chinese Medical Association (29). The clustering of CV risk factors was defined as the sum of the number of risk factors (abdominal obesity, elevated BP, high FBG, high TG and low HDL-C) with the range spanning from 0 to 4. As only few children had more than 3 risk factors, we combined those with 3–4 risk factors into a same group as ≥3, and the clustering was further categorized as 0, 1, 2 and ≥3. Additionally, we re-defined these CV risk factors according to the same distribution of the present study population, namely the corresponding 90th, 85th and 80th sex- and age-specific percentile values of WC, systolic/diastolic BP, FBG and TG, and 10th, 15th and 20th percentile values of HDL-C.

LVH was defined as LVMI ≥90th percentile values for sex and age of this population, and high RWT was defined as RWT ≥90th percentile values for sex and age of this population (the sex- and age-specific percentile cutoffs of LVMI and RWT are provided in Supplementary Table S1). LVG patterns were defined as normal geometry (normal LVMI and normal RWT), CR (normal LVMI and high RWT), EH (LVH and normal RWT) and CH (LVH and high RWT).

### 2.5. Statistical analysis

All statistical analyses were performed using SAS software version 9.4 (SAS Institute, Cary, NC, USA), and a two-sided *P* < 0.05 was considered statistically significant. Continuous variables were presented as means ± standard deviations and categorical variables as numbers (percentages). Students' *t* test and chi-square test were used to examine differences in characteristics by the LVH status (presence vs. absence) or the RWT status (high vs. normal). Pearson correlation analysis was performed between single CV risk factors and LVMI and RWT. Potential covariates used for adjustment included sex, age, sleep duration (<9 vs. ≥9 h per day), screen time (≤2 vs. >2 h per day), physical activity (<1 vs. ≥1 h per day), and intake of vegetables and fruits (<5 vs. ≥5 servings per day). Adjusted LVMI and RWT levels between each CV risk factor status (abnormal vs. normal) and across the number of CV risk factors (0, 1, 2 and ≥3) were compared using covariance analysis. We also conducted trend analysis for LVMI and RWT levels according to the number of CV risk factors using multivariate linear regression analysis. Binary logistic regression analysis was used to calculate adjusted odds ratios (ORs) and 95% confidence intervals (CIs) of LVH and high RWT, and multi-class logistic regression analysis for LVG remodeling with the normal geometry as the reference category.

## 3. Results

### 3.1. Characteristics of study participants

A total of 1,406 children (boys: 52.8%) aged 6–11 years were included in this study. Characteristics of children by the LVH status are shown in Table 1. Children with LVH (*n* = 133) had higher levels of BMI, WC, systolic and diastolic BP, and TG, and a lower level of HDL-C than those without LVH (*n* = 1,273). Similar characteristics were found between children with high RWT and those with normal RWT (Supplementary Table S2).

**Table 1:** Characteristics of the children according to the LVH status.

Characteristics	Total (*n* = 1,406)	LVH status		
		Presence (*n* = 133)	Absence (*n* = 1,273)	*P* value^a^
Age, years	8.93 ± 1.50	8.87 ± 1.53	8.93 ± 1.50	0.613
Height, cm	136.40 ± 10.67	135.73 ± 12.72	136.46 ± 10.44	0.524
Body mass index, kg/m^2^	18.21 ± 3.46	22.54 ± 4.41	17.76 ± 3.01	<0.001
Waist circumference, cm	63.03 ± 9.78	73.05 ± 12.94	61.98 ± 8.75	<0.001
Systolic BP, mmHg	106.43 ± 9.20	108.68 ± 9.75	106.19 ± 9.11	0.003
Diastolic BP, mmHg	63.60 ± 6.66	65.31 ± 7.47	63.42 ± 6.55	0.006
FBG, mmol/L	4.73 ± 0.56	4.81 ± 0.59	4.72 ± 0.55	0.104
TG, mmol/L	0.76 ± 0.35	0.97 ± 0.47	0.74 ± 0.32	<0.001
HDL-C, mmol/L	1.58 ± 0.38	1.44 ± 0.36	1.60 ± 0.38	<0.001
Abdominal obesity, *n* (%)	440 (31.29)	96 (72.18)	344 (27.02)	<0.001
Elevated BP, *n* (%)	215 (15.29)	28 (21.05)	187 (14.69)	0.052
High FBG, *n* (%)	88 (6.26)	15 (11.28)	73 (5.73)	0.012
High TG, *n* (%)	64 (4.55)	19 (14.29)	45 (3.53)	<0.001
Low HDL-C, *n* (%)	76 (5.41)	16 (12.03)	60 (4.71)	<0.001
Short sleep duration, *n* (%)	229 (16.29)	11 (8.27)	218 (17.12)	0.009
Long screen time, *n* (%)	62 (4.41)	9 (6.77)	53 (4.16)	0.164
Insufficient physical activity, *n* (%)	796 (56.61)	78 (58.65)	718 (56.40)	0.619
Insufficient intake of fruits and/or vegetables, *n* (%)	1,142 (81.22)	115 (86.47)	1,027 (80.68)	0.104

Continuous variables are presented as means ± standard deviations. BP, blood pressure; FBG, fasting blood glucose; HDL-C, high-density lipoprotein cholesterol; LVH, left ventricular hypertrophy; TG, triglycerides.

^a^ Group differences between the LVH status.

### 3.2. Association of single CV risk factor with LVG remodeling

Abdominal obesity, high TG and low HDL-C were associated with the risk of LVH irrespective of definitions of CV risk factors (Table 2). Elevated BP was associated with LVH when using percentile values of this population (Table 2). High FBG was associated with LVH when using recommended guideline (Table 2). Similarly, regardless of definitions of CV risk factors, abdominal obesity and high TG were associated with increased risk of high RWT, while associations of elevated BP, high FBG and low HDL-C with high RWT varied slightly based on different definitions of CV risk factors (Table 2). Besides, there were positive correlations between specific CV risk factors (including WC, SBP, DBP, FBG, and TG; Supplementary Table S3) and LVMI and RWT, and HDL-C was related inversely with LVMI (*r* = −0.12, *P* < 0.001; Supplementary Table S3). Additionally, covariance analysis showed significant associations of each CV risk factor with LVMI and RWT levels (Supplementary Table S4).

**Table 2:** Associations of each CV risk factor with LVH and high RWT.

CV risk factors	LVH			High RWT		
	*n* (%)^b^	OR (95% CI)	*P* value	*n* (%)^b^	OR (95% CI)	*P* value
Abdominal obesity						
Recommended^a^ (*n* = 440)	96 (21.82)	6.85 (4.58–10.24)	<0.001	79 (17.95)	3.42 (2.38–4.91)	<0.001
P90 (*n* = 146)	53 (36.30)	8.52 (5.62–12.93)	<0.001	37 (25.34)	4.10 (2.66–6.33)	<0.001
P85 (*n* = 216)	68 (31.48)	8.10 (5.49–11.95)	<0.001	53 (24.54)	4.40 (2.99–6.47)	<0.001
P80 (*n* = 286)	84 (29.37)	9.08 (6.16–13.39)	<0.001	65 (22.73)	4.26 (2.95–6.15)	<0.001
Elevated BP						
Recommended^a^ (*n* = 215)	28 (13.02)	1.48 (0.95–2.32)	0.087	26 (12.09)	1.31 (0.83–2.07)	0.240
P90 (*n* = 234)	34 (14.53)	1.77 (1.16–2.69)	0.008	37 (15.81)	2.00 (1.33–3.00)	0.001
P85 (*n* = 341)	44 (12.90)	1.57 (1.07–2.31)	0.022	48 (14.08)	1.75 (1.20–2.55)	0.003
P80 (*n* = 446)	53 (11.88)	1.46 (1.01–2.11)	0.044	56 (12.56)	1.50 (1.05–2.16)	0.026
High FBG						
Recommended^a^ (*n* = 88)	15 (17.05)	2.21 (1.21–4.06)	0.010	15 (17.05)	1.94 (1.06–3.53)	0.031
P90 (*n* = 148)	19 (12.84)	1.52 (0.90–2.56)	0.118	20 (13.51)	1.48 (0.89–2.46)	0.132
P85 (*n* = 217)	25 (11.52)	1.34 (0.84–2.14)	0.219	30 (13.82)	1.56 (1.01–2.42)	0.044
P80 (*n* = 288)	34 (11.81)	1.43 (0.94–2.18)	0.091	41 (14.24)	1.71 (1.15–2.53)	0.008
High TG						
Recommended^a^ (*n* = 64)	19 (29.69)	5.15 (2.83–9.38)	<0.001	12 (18.75)	2.18 (1.12–4.26)	0.022
P90 (*n* = 150)	32 (21.33)	3.10 (1.99–4.84)	<0.001	25 (16.67)	1.98 (1.23–3.17)	0.005
P85 (*n* = 220)	42 (19.09)	2.88 (1.93–4.31)	<0.001	33 (15.00)	1.80 (1.18–2.74)	0.007
P80 (*n* = 291)	54 (18.56)	3.02 (2.07–4.40)	<0.001	41 (14.09)	1.71 (1.15–2.52)	0.008
Low HDL-C						
Recommended^a^ (*n* = 76)	16 (21.05)	2.90 (1.61–5.25)	<0.001	16 (21.05)	2.71 (1.51–4.86)	0.001
P10 (*n* = 132)	27 (20.45)	2.89 (1.80–4.63)	<0.001	19 (14.39)	1.67 (0.99–2.82)	0.055
P15 (*n* = 200)	31 (15.50)	2.03 (1.31–3.15)	0.002	25 (12.50)	1.41 (0.89–2.25)	0.143
P20 (*n* = 277)	40 (14.44)	1.90 (1.28–2.83)	0.002	37 (13.36)	1.57 (1.05–2.35)	0.028

Binary logistic regression models were performed separately for each cardiovascular risk factor adjusting for sex, age, sleep duration, screen time, physical activity, and intake of vegetables and fruits. P90, P85 and P80 represented the corresponding sex- and age-specific percentile values of waist circumference, systolic/diastolic BP, FBG and TG based on the present population, and P10, P15 and P20 for HDL-C. BP, blood pressure; CI, confidence interval; CV, cardiovascular; FBG, fasting blood glucose; HDL-C, high-density lipoprotein cholesterol; LVH, left ventricular hypertrophy; OR, odds ratio; RWT, relative wall thickness; TG, triglycerides.

^a^ Recommended guideline from the Society of Pediatrics, Chinese Medical Association.

^b^ Percentages are calculated as: (the number of subjects with LVH or high RWT)/(the number of those with specific CV risk factors)×100%.

Abdominal obesity (OR = 2.34, 95% CI: 1.53–3.59) and low HDL-C (OR = 2.36, 95% CI: 1.12–4.97) were associated with CR. Elevated BP (90th or 85th) and high TG (85th) defined using the percentiles were also associated with CR (Supplementary Table S5). Abdominal obesity, elevated BP, high TG, and low HDL-C increased the risk of EH with ORs (95% CIs) of 5.26 (3.34–8.29), 1.72 (1.03–2.89), 5.92 (2.92–11.98), and 2.58 (1.21–5.47), respectively; these associations were mostly similar when defining CV risk factors based on the percentiles (Supplementary Table S5). Abdominal obesity, high FBG, high TG, and low HDL-C also increased the risk of CH, with ORs (95% CIs) of 20.96 (8.13–54.03), 3.33 (1.38–8.06), 4.82 (1.86–12.52), and 4.58 (1.92–10.92), respectively; similar associations were found when CV risk factors were defined using the percentiles for definitions (Supplementary Table S5).

### 3.3. Association of clustering of CV risk factors with LVG remodeling

**Figure 1:**
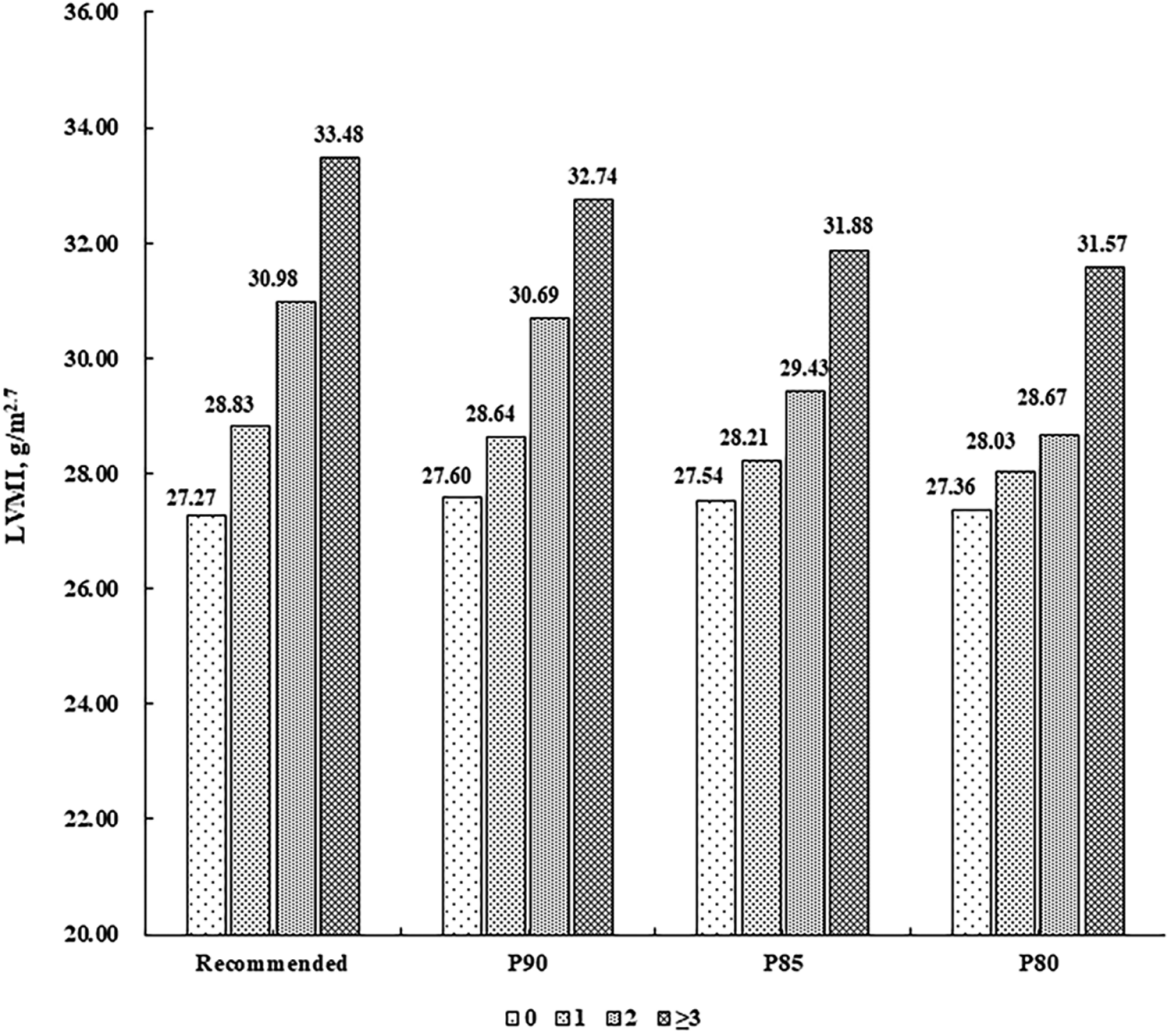
Mean levels of LVMI (g/m^2.7^) according to the number of cardiovascular risk factors. Cardiovascular risk factors were defined according to the recommended guideline from the Society of Pediatrics, Chinese Medical Association and the corresponding sex- and age-specific percentile values based on the present population, respectively (P90, P85 and P80 for waist circumference, systolic/diastolic blood pressure, fasting blood glucose, and triglycerides, and P10, P15 and P20 for high-density lipoprotein cholesterol). LVMI, left ventricular mass index.

**Figure 2:**
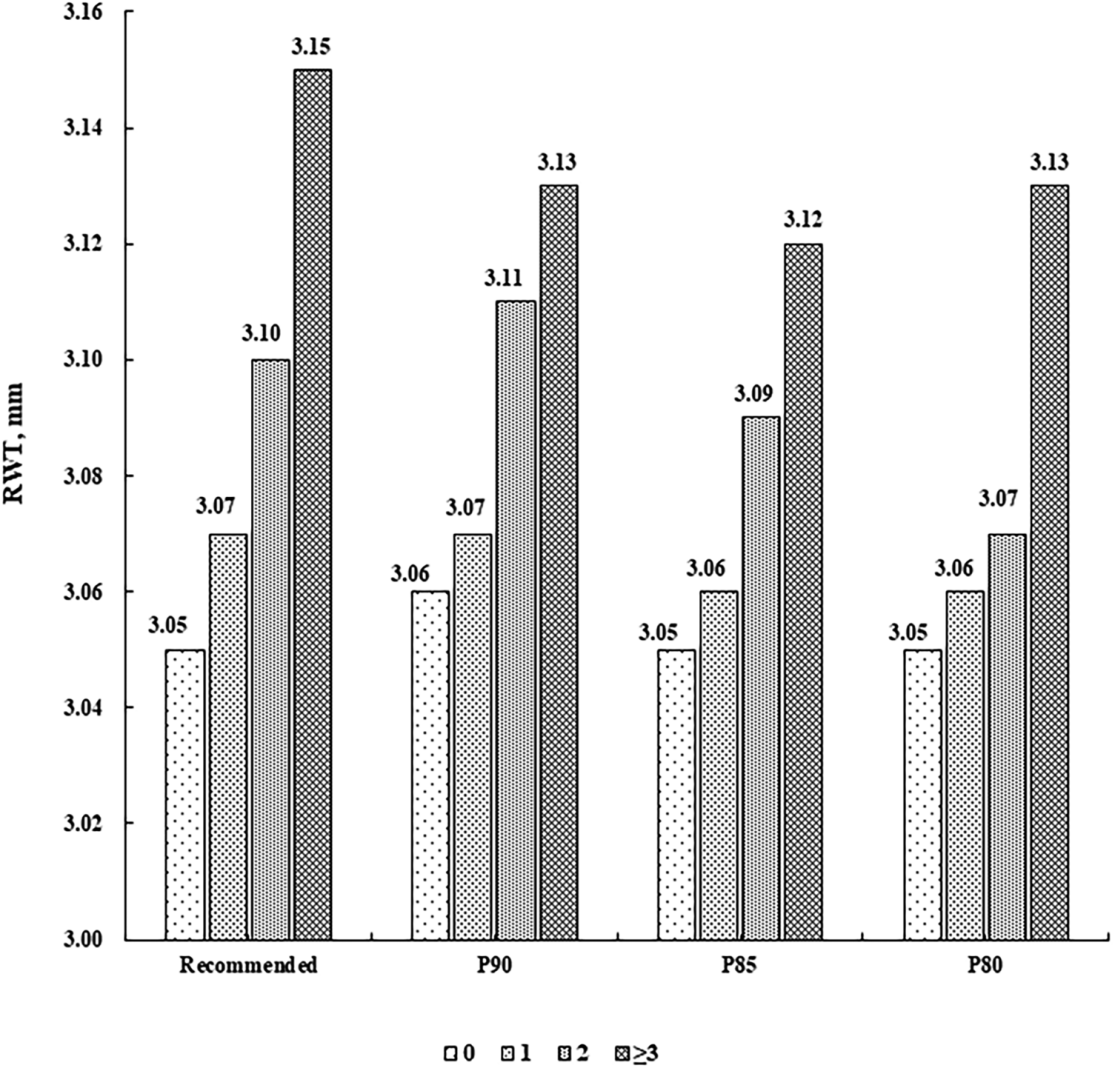
Mean levels of RWT (mm) according to the number of cardiovascular risk factors. Cardiovascular risk factors were defined according to the recommended guideline from the Society of Pediatrics, Chinese Medical Association and the corresponding sex- and age-specific percentile values based on the present population, respectively (P90, P85 and P80 for waist circumference, systolic/diastolic blood pressure, fasting blood glucose, and triglycerides, and P10, P15 and P20 for high-density lipoprotein cholesterol). RWT, relative wall thickness.

There were 787 (55.97%) children without CV risk factor, 413 (29.37%) with one, 156 (11.10%) with two, and 50 (3.56%) with at least three. LVMI and RWT levels were higher along increasing number of CV risk factors (*P* for linear trend <0.001), after adjustment for sex, age, sleep duration, screen time, physical activity, and intake of vegetables and fruits (Figures 1, 2).

The prevalence of LVH and high RWT increased progressively across the clustering of CV risk factors (Table 3). Compared with children without CV risk factor, those with 1, 2 and ≥3 CV risk factors were associated with an increased risk of LVH, with ORs (95% CIs) of 3.49 (2.19–5.56), 5.53 (3.20–9.55), and 19.19 (9.67–38.08), respectively (Table 3). Similarly, the more number of CV risk factors was associated with a higher risk of high RWT [2.47 (1.63–3.74) for 1, 3.76 (2.25–6.27) for 2, and 5.47 (2.65–11.28) for ≥3, Table 3]. Similar associations were found when CV risk factors were defined based on the percentiles (Table 3).

**Table 3:** Associations of the clustering of CV risk factors with LVH and high RWT.

Cut-offs	Number of CV risk factors	LVH		High RWT	
		*n* (%)	OR (95% CI)	*n* (%)	OR (95% CI)
Recommended^a^	0 (*n* = 787)	31 (3.94)	Reference	45 (5.72)	Reference
	1 (*n* = 413)	52 (12.59)	3.49 (2.19–5.56)	54 (13.08)	2.47 (1.63–3.74)
	2 (*n* = 156)	29 (18.59)	5.53 (3.20–9.55)	28 (17.95)	3.76 (2.25–6.27)
	≥3 (*n* = 50)	21 (42.00)	19.19 (9.67–38.08)	12 (24.00)	5.47 (2.65–11.28)
P90	0 (*n* = 853)	42 (4.92)	Reference	53 (6.21)	Reference
	1 (*n* = 367)	43 (11.72)	2.54 (1.62–3.96)	49 (13.35)	2.34 (1.56–3.54)
	2 (*n* = 129)	27 (20.93)	5.11 (3.00–8.69)	26 (20.16)	3.89 (2.32–6.52)
	≥3 (*n* = 57)	21 (36.84)	11.19 (5.96–20.98)	11 (19.30)	3.73 (1.82–7.65)
P85	0 (*n* = 684)	32 (4.68)	Reference	40 (5.85)	Reference
	1 (*n* = 398)	35 (8.79)	2.03 (1.23–3.34)	43 (10.80)	1.95 (1.25–3.07)
	2 (*n* = 209)	31 (14.83)	3.43 (2.03–5.79)	30 (14.35)	2.69 (1.62–4.45)
	≥3 (*n* = 115)	35 (30.43)	9.23 (5.38–15.83)	26 (22.61)	4.87 (2.83–8.39)
P80	0 (*n* = 529)	18 (3.40)	Reference	31 (5.86)	Reference
	1 (*n* = 413)	35 (8.47)	2.78 (1.55–5.01)	34 (8.23)	1.42 (0.86–2.36)
	2 (*n* = 282)	27 (9.57)	2.96 (1.59–5.49)	34 (12.06)	2.20 (1.32–3.68)
	≥3 (*n* = 182)	53 (29.12)	12.08 (6.81–21.44)	40 (21.98)	4.50 (2.71–7.47)

Binary logistic regression models were performed with adjustment for sex, age, sleep duration, screen time, physical activity, and intake of vegetables and fruits. P90, P85 and P80 represented the corresponding sex- and age-specific percentile values of waist circumference, systolic/diastolic blood pressure, fasting blood glucose and triglycerides based on the present population, and P10, P15 and P20 for high-density lipoprotein cholesterol. CI, confidence interval; CV, cardiovascular; LVH, left ventricular hypertrophy; OR, odds ratio; RWT, relative wall thickness.

^a^ Recommended guideline from the Society of Pediatrics, Chinese Medical Association.

The clustering of CV risk factors was associated with LVG remodeling including CR, EH and CH (Table 4). The corresponding ORs (95% CIs) of CR were 1.71 (1.06–2.76), 2.83 (1.54–5.18), and 3.82 (1.37–10.62), respectively, for children with 1, 2 and ≥3 CV risk factors; those of EH were 2.42 (1.42–4.11), 4.23 (2.24–7.96), and 16.86 (7.70–36.92), respectively; those of CH were 14.92 (4.41–50.47), 23.15 (6.32–84.83), and 71.19 (17.09–296.56), respectively. Similar associations of the clustering of CV risk factors with CR, EH and CH were found when using the percentiles for definitions.

**Table 4:** Association of the clustering of CV risk factors with LVG.

Cut-offs	Number of CV risk factors	CR		EH		CH	
		*n* (%)	OR (95% CI)	*n* (%)	OR (95% CI)	*n* (%)	OR (95% CI)
Recommended^a^	0 (*n* = 787)	42 (5.34)	Reference	28 (3.56)	Reference	3 (0.38)	Reference
	1 (*n* = 413)	33 (7.99)	1.71 (1.06–2.76)	31 (7.51)	2.42 (1.42–4.11)	21 (5.08)	14.92 (4.41–50.47)
	2 (*n* = 156)	17 (10.90)	2.83 (1.54–5.18)	18 (11.54)	4.23 (2.24–7.96)	11 (7.05)	23.15 (6.32–84.83)
	≥3 (*n* = 50)	5 (10.00)	3.82 (1.37–10.62)	14 (28.00)	16.86 (7.70–36.92)	7 (14.00)	71.19 (17.09–296.56)
P90	0 (*n* = 853)	46 (5.39)	Reference	35 (4.10)	Reference	7 (0.82)	Reference
	1 (*n* = 367)	31 (8.45)	1.79 (1.11–2.89)	25 (6.81)	1.85 (1.09–3.16)	18 (4.90)	6.63 (2.74–16.05)
	2 (*n* = 129)	13 (10.08)	2.55 (1.32–4.92)	14 (10.85)	3.52 (1.81–6.84)	13 (10.08)	15.46 (5.97–40.02)
	≥3 (*n* = 57)	7 (12.28)	4.33 (1.79–10.48)	17 (29.82)	13.12 (6.52–26.41)	4 (7.02)	14.49 (3.99–52.65)
P85	0 (*n* = 684)	34 (4.97)	Reference	26 (3.80)	Reference	6 (0.88)	Reference
	1 (*n* = 398)	31 (7.79)	1.70 (1.03–2.83)	23 (5.78)	1.72 (0.97–3.07)	12 (3.02)	3.77 (1.40–10.15)
	2 (*n* = 209)	19 (9.09)	2.23 (1.23–4.03)	20 (9.57)	2.95 (1.60–5.45)	11 (5.26)	6.62 (2.40–18.28)
	≥3 (*n* = 115)	13 (11.30)	3.71 (1.86–7.41)	22 (19.13)	8.23 (4.39–15.45)	13 (11.30)	20.24 (7.41–55.30)
P80	0 (*n* = 529)	27 (5.10)	Reference	14 (2.65)	Reference	4 (0.76)	Reference
	1 (*n* = 413)	26 (6.30)	1.30 (0.74–2.27)	27 (6.54)	2.85 (1.47–5.53)	8 (1.94)	2.81 (0.84–9.44)
	2 (*n* = 282)	27 (9.57)	2.19 (1.25–3.84)	20 (7.09)	3.06 (1.51–6.19)	7 (2.48)	3.48 (1.00–12.06)
	≥3 (*n* = 182)	17 (9.34)	2.70 (1.42–5.15)	30 (16.48)	9.75 (4.97–19.10)	23 (12.64)	24.82 (8.39–73.46)

Multi-class logistic regression models were performed with adjustment for sex, age, sleep duration, screen time, physical activity, and intake of vegetables and fruits. P90, P85 and P80 represented the corresponding sex- and age-specific percentile values of waist circumference, systolic/diastolic blood pressure, fasting blood glucose and triglycerides based on the present population, and P10, P15 and P20 for high-density lipoprotein cholesterol. CH, concentric hypertrophy; CI, confidence interval; CR, concentric remodeling; CV, cardiovascular; EH, eccentric hypertrophy; LVG, left ventricular geometric; OR, odds ratio.

^a^ Recommended guideline from the Society of Pediatrics, Chinese Medical Association.

## 4. Discussion

In this cross-sectional study among 1,406 children aged 6–11 years from China, we found that increased numbers of CV risk factors were associated with the higher levels of LVMI and RWT. The clustering of CV risk factors progressively increased the risk of LVH, high RWT and LVG remodeling (CR, EH and CH). These findings indicate a strong association between the number of CV risk factors and the risk of LVG remodeling among children.

Consistent with previous studies, we found that specific CV risk factors were significantly associated with LVMI and RWT. A retrospective study including 160 overweight and obese children and adolescents aged 6–18 years found that WC above the 90th percentile values was associated with increased LVMI and CH (33). A cross-sectional study of 303 adolescents (mean age 15.6 years) showed an independent relationship between BP and LVMI (9). A cross-sectional study of 70 children with obesity (median age 14 years) found positive correlations of TG and the TG-to-HDL-C ratio with LVMI and RWT (15). Associations of the TG-to-HDL-C ratio with LVMI and RWT were also found in a study including 884 outpatient children and adolescents aged 6–16 years (16). In the present study, WC, SBP, DBP, FBG, and TG were positively related with LVMI and RWT, along with inverse correlation between HDL-C and LVMI. Our findings, combined with prior research, may suggest that monitoring CV risk factors among children holds practical value for the prevention of subclinical target organ damage.

Comparatively, abdominal obesity among all five CV risk factors had the largest effect on the cardiac remodeling. Obesity has been considered as a state of chronic metabolic disorder (34), and obesity-related hemodynamic factors (e.g., increased circulating blood volume and cardiac output) and metabolic factors (e.g., insulin resistance, visceral fat deposition and secretion of adipokines) may contribute to a series of adaptations/alterations in cardiac structure and function (35). Besides, obesity frequently promotes other CV risk factors such as hypertension, dyslipidemia, and glucose intolerance (36), and there is also an additive effect between obesity and BP on cardiac remodeling (37). Evidence shows that elevated BP is a prominent contributor to cardiac remodeling (38), however, a weaker association between elevated BP (vs. abdominal obesity) and cardiac remodeling was found in our study. This may suggest that other underlying mechanisms other than BP-related responses may play a vital role. Further studies are warranted to clarify the pathophysiologic mechanisms.

Furthermore, our study also showed that LVMI and RWT levels increased markedly according to the number of CV risk factors. Similarly, a cross-sectional study of 830 young US adults aged 24–43 years found that LVMI levels increased according to the number of abnormal MetS components (39). In another cross-sectional study of 291 children (mean age 11.7 years), a comprehensive CV risk score based on five MetS components had a positive correlation with cIMT levels (40). Overall, these findings support an additive effect of these risk factors on continuous subclinical CVD markers.

A recent combined analysis of three population-based studies of 2,427 children and adolescents aged 6–17 years showed that a graded risk score based on five MetS components was superior to dichotomous classification of MetS to predict high cIMT (22). Additionally, our findings that having ≥3 CV risk factors was associated with the highest risk of LVG remodeling, followed by having two and one CV risk factors, indicate a dose-response association between the clustering of CV risk factors and LVG remodeling. Of note, children who did not meet the definition of MetS (i.e., number of CV risk factors as 1 or 2) were also at an increased risk of LVG remodeling, re-emphasizing the continuous nature of the association between CV risk factors and cardiac outcomes. Therefore, our findings supported that it is important to focus on clustering of CV risk factors among children and adolescents rather than the arbitrarily defined MetS (20). Moreover, irrespective of what are cutoffs of CV risk factors, it is essential to keep an optimal levels of health factors. As exemplified in a child cohort study from China, the ideal levels of four behavior factors (i.e., diet, physical activity, nicotine exposure, sleep heath) as well as four health indicators (i.e., BMI, blood lipids, blood glucose, and blood pressure) were conducive to reducing the abnormal cardiovascular structures (41).

To our knowledge, this is the first study examining the association between the clustering of CV risk factors and LVG remodeling among Chinese children. Meanwhile, there are several limitations in this study that should be considered. First, this study included children aged 6–11 years from only one primary school, making our results less generalizable. Further studies in children with different ages from other regions are needed to further validate the associations observed in this study. Second, the cross-sectional data used in this study preclude concluding on the causal link of CV risk factors on cardiac outcomes. Third, although different CV risk factors had different effects on the cardiac structure, and we did not allocate different weights to specific risk factors because of unavailable weights, we partially addressed this issue by considering these CV risk factors along percentile values of the present population.

## 5. Conclusion

CV risk factors in isolation and combination were associated with an increased risk of LVH, high RWT and LVG remodeling among children, emphasizing the need to consider multiple risk factors when assessing the risk of cardiac outcomes.

## Data Availability

The raw data supporting the conclusions of this article will be made available by the authors, without undue reservation.
