# Supplementary material for: Association between clustering of cardiovascular risk factors and left ventricular geometric remodeling in Chinese children
Source: Front Cardiovasc Med. 2023 Aug 17;10:1236730. doi: 10.3389/fcvm.2023.1236730 (PMC10469610; doi:10.3389/fcvm.2023.1236730)
Supplement: Supplementary file 1 [file Table1.doc]

**Table S1. Sex- and age-specific 90th percentile values of LVMI and RWT among children aged 6-11 years**

| Sex | Age, year | LVMI, g/m^2.7^ | RWT, mm |
| --- | --- | --- | --- |
| Boy | 6 | 33.52882 | 0.30763 |
|  | 7 | 33.21138 | 0.31212 |
|  | 8 | 33.61655 | 0.32709 |
|  | 9 | 36.70782 | 0.33589 |
|  | 10 | 38.89584 | 0.35000 |
|  | 11 | 39.32212 | 0.35293 |
| Girl | 6 | 34.44436 | 0.30593 |
|  | 7 | 32.66745 | 0.31063 |
|  | 8 | 29.01595 | 0.31212 |
|  | 9 | 31.27172 | 0.32689 |
|  | 10 | 31.79859 | 0.33822 |
|  | 11 | 32.26162 | 0.33570 |

LVMI, left ventricular mass index; RWT, relative wall thickness.

**Table S2. Characteristics of the children according to the RWT status**

| Characteristics | Total (n=1406) | RWT status | | |
| --- | --- | --- | --- | --- |
|  |  | High (n=139) | Normal (n=1267) | *P* value^*^ |
| Age, years | 8.93±1.50 | 9.00±1.50 | 8.92±1.50 | 0.531 |
| Height, cm | 136.4±10.67 | 139.11±10.40 | 136.10±10.66 | 0.002 |
| Body mass index, kg/m^2^ | 18.21±3.46 | 20.44±4.23 | 17.97±3.27 | <0.001 |
| Waist circumference, cm | 63.03±9.78 | 69.05±11.17 | 62.37±9.39 | <0.001 |
| Systolic BP, mmHg | 106.43±9.20 | 108.46±8.37 | 106.21±9.26 | 0.006 |
| Diastolic BP, mmHg | 63.60±6.66 | 64.72±6.79 | 63.48±6.64 | 0.037 |
| FBG, mmol/L | 4.73±0.56 | 4.83±0.63 | 4.72±0.55 | 0.041 |
| TG, mmol/L | 0.76±0.35 | 0.89±0.42 | 0.75±0.33 | <0.001 |
| HDL-C, mmol/L | 1.58±0.38 | 1.46±0.34 | 1.59±0.38 | <0.001 |
| Abdominal obesity, n (%) | 440 (31.29) | 79 (56.83) | 361 (28.49) | <0.001 |
| Elevated BP, n (%) | 215 (15.29) | 26 (18.71) | 189 (14.92) | 0.239 |
| High FBG, n (%) | 88 (6.26) | 15 (10.79) | 73 (5.76) | 0.020 |
| High TG, n (%) | 64 (4.55) | 12 (8.63) | 52 (4.10) | 0.015 |
| Low HDL-C, n (%) | 76 (5.41) | 16 (11.51) | 60 (4.74) | 0.001 |
| Short sleep duration, n (%) | 229 (16.29) | 17 (12.23) | 212 (16.73) | 0.172 |
| Long screen time, n (%) | 62 (4.41) | 7 (5.04) | 55 (4.34) | 0.705 |
| Insufficient physical activity, n (%) | 796 (56.61) | 74 (53.24) | 722 (56.99) | 0.397 |
| Insufficient intake of fruits and/or vegetables, n (%) | 1142 (81.22) | 107 (76.98) | 1035 (81.69) | 0.177 |

Continuous variables are presented as means ± standard deviations.

^*^Group difference between the RWT status.

BP, blood pressure; FBG, fasting blood glucose; HDL-C, high-density lipoprotein cholesterol; RWT, relative wall thickness; TG, triglycerides.

**Table S3. Correlations between each CV risk factor and LVMI and RWT**

| CV risk factors | LVMI, g/m^2.7^ | |  | RWT, mm | |
| --- | --- | --- | --- | --- | --- |
|  | *r* | *P* value |  | *r* | *P* value |
| Waist circumference | 0.44 | <0.001 |  | 0.37 | <0.001 |
| Systolic BP | 0.07 | 0.008 |  | 0.22 | <0.001 |
| Diastolic BP | 0.03 | 0.200 |  | 0.12 | <0.001 |
| FBG | 0.09 | <0.001 |  | 0.23 | <0.001 |
| TG | 0.21 | <0.001 |  | 0.23 | <0.001 |
| HDL-C | -0.12 | <0.001 |  | -0.05 | 0.073 |

BP, blood pressure; CV, cardiovascular; FBG, fasting blood glucose; HDL-C, high-density lipoprotein cholesterol; LVMI, left ventricular mass index; RWT, relative wall thickness; TG, triglycerides.

**Table S4.** **Associations of each CV risk factor with LVMI and RWT**

| CV risk factors | LVMI, g/m^2.7^ |  | RWT, mm |
| --- | --- | --- | --- |
| **Abdominal obesity** |  |  |  |
| Recommended^*^ |  |  |  |
| No | 27.33 (27.05-27.60) |  | 3.05 (3.04-3.06) |
| Yes | 30.63 (30.23-31.04) |  | 3.10 (3.09-3.12) |
| *P* value | <0.001 |  | <0.001 |
| P90 |  |  |  |
| No | 27.84 (27.60-28.08) |  | 3.06 (3.05-3.07) |
| Yes | 32.86 (32.15-33.57) |  | 3.14 (3.12-3.17) |
| *P* value | <0.001 |  | <0.001 |
| P85 |  |  |  |
| No | 27.66 (27.42-27.91) |  | 3.06 (3.05-3.07) |
| Yes | 32.22 (31.64-32.80) |  | 3.13 (3.11-3.15) |
| *P* value | <0.001 |  | <0.001 |
| P80 |  |  |  |
| No | 27.49 (27.24-27.74) |  | 3.05 (3.04-3.06) |
| Yes | 31.77 (31.27-32.27) |  | 3.13 (3.11-3.15) |
| *P* value | <0.001 |  | <0.001 |
| **Elevated BP** |  |  |  |
| Recommended^*^ |  |  |  |
| No | 28.21 (27.95-28.47) |  | 3.07 (3.06-3.08) |
| Yes | 29.21 (28.59-29.83) |  | 3.07 (3.05-3.10) |
| *P* value | 0.004 |  | 0.529 |
| P90 |  |  |  |
| No | 28.20 (27.94-28.46) |  | 3.06 (3.05-3.07) |
| Yes | 29.18 (28.59-29.77) |  | 3.09 (3.06-3.11) |
| *P* value | 0.003 |  | 0.061 |
| P85 |  |  |  |
| No | 28.21 (27.93-28.48) |  | 3.06 (3.05-3.07) |
| Yes | 28.86 (28.37-29.35) |  | 3.09 (3.07-3.10) |
| *P* value | 0.023 |  | 0.020 |
| P80 |  |  |  |
| No | 28.21 (27.92-28.50) |  | 3.06 (3.05-3.07) |
| Yes | 28.70 (28.27-29.12) |  | 3.08 (3.07-3.10) |
| *P* value | 0.064 |  | 0.026 |
| **High FBG** |  |  |  |
| Recommended^*^ |  |  |  |
| No | 28.25 (28.00-28.50) |  | 3.06 (3.06-3.07) |
| Yes | 30.10 (29.12-31.07) |  | 3.11 (3.07-3.14) |
| *P* value | <0.001 |  | 0.027 |
| P90 |  |  |  |
| No | 28.25 (28.00-28.51) |  | 3.06 (3.05-3.07) |
| Yes | 29.32 (28.58-30.06) |  | 3.09 (3.06-3.12) |
| *P* value | 0.008 |  | 0.063 |
| P85 |  |  |  |
| No | 28.23 (27.97-28.50) |  | 3.06 (3.05-3.07) |
| Yes | 29.07 (28.46-29.69) |  | 3.09 (3.06-3.11) |
| *P* value | 0.014 |  | 0.056 |
| P80 |  |  |  |
| No | 28.20 (27.93-28.47) |  | 3.06 (3.05-3.07) |
| Yes | 28.98 (28.45-29.52) |  | 3.09 (3.07-3.10) |
| *P* value | 0.011 |  | 0.041 |
| **High TG** |  |  |  |
| Recommended^*^ |  |  |  |
| No | 28.15 (27.91-28.40) |  | 3.07 (3.06-3.08) |
| Yes | 32.75 (31.63-33.86) |  | 3.09 (3.05-3.13) |
| *P* value | <0.001 |  | 0.291 |
| P90 |  |  |  |
| No | 28.12 (27.86-28.37) |  | 3.06 (3.06-3.07) |
| Yes | 30.45 (29.72-31.18) |  | 3.08 (3.06-3.11) |
| *P* value | <0.001 |  | 0.187 |
| P85 |  |  |  |
| No | 28.07 (27.81-28.33) |  | 3.06 (3.05-3.07) |
| Yes | 29.94 (29.34-30.54) |  | 3.08 (3.06-3.11) |
| *P* value | <0.001 |  | 0.114 |
| P80 |  |  |  |
| No | 28.00 (27.73-28.27) |  | 3.06 (3.05-3.07) |
| Yes | 29.76 (29.24-30.29) |  | 3.08 (3.06-3.10) |
| *P* value | <0.001 |  | 0.082 |
| **Low HDL-C** |  |  |  |
| Recommended^*^ |  |  |  |
| No | 28.23 (27.98-28.47) |  | 3.06 (3.05-3.07) |
| Yes | 30.72 (29.69-31.75) |  | 3.12 (3.08-3.16) |
| *P* value | <0.001 |  | 0.007 |
| P10 |  |  |  |
| No | 28.20 (27.95-28.45) |  | 3.07 (3.06-3.07) |
| Yes | 29.96 (29.17-30.74) |  | 3.08 (3.05-3.11) |
| *P* value | <0.001 |  | 0.361 |
| P15 |  |  |  |
| No | 28.20 (27.94-28.46) |  | 3.07 (3.06-3.08) |
| Yes | 29.33 (28.7-29.97) |  | 3.07 (3.05-3.09) |
| *P* value | 0.001 |  | 0.875 |
| P20 |  |  |  |
| No | 28.12 (27.85-28.39) |  | 3.07 (3.06-3.08) |
| Yes | 29.36 (28.82-29.90) |  | 3.07 (3.05-3.09) |
| *P* value | <0.001 |  | 0.827 |

Data are expressed as means (95% confidence intervals) after adjusting for sex, age, sleep duration, screen time, physical activity, and intake of vegetables and fruits.

^*^Recommended guideline from the Society of Pediatrics, Chinese Medical Association.

P90, P85 and P80 represented the corresponding sex- and age-specific percentile values of waist circumference, systolic/diastolic BP, FBG and TG based on the present population, and P10, P15 and P20 for HDL-C.

BP, blood pressure; CV, cardiovascular; FBG, fasting blood glucose; HDL-C, high-density lipoprotein cholesterol; LVMI, left ventricular mass index; RWT, relative wall thickness; TG, triglycerides.

**Table S5. Association of each CV risk factor with LVG**

| CV risk factors | CR | |  | EH | |  | CH | |
| --- | --- | --- | --- | --- | --- | --- | --- | --- |
|  | n (%)† | *OR* (95% *CI*) |  | n (%)† | *OR* (95% *CI*) |  | n (%)† | *OR* (95% *CI*) |
| **Abdominal obesity** |  |  |  |  |  |  |  |  |
| Recommended^*^(n=440) | 42 (9.55) | 2.34 (1.53-3.59) |  | 59 (13.41) | 5.26 (3.34-8.29) |  | 37 (8.41) | 20.96 (8.13-54.03) |
| P90 (n=146) | 19 (13.01) | 4.08 (2.32-7.18) |  | 35 (23.97) | 9.86 (5.99-16.24) |  | 18 (12.33) | 10.95 (5.59-21.43) |
| P85 (n=216) | 27 (12.50) | 3.72 (2.28-6.07) |  | 42 (19.44) | 7.91 (4.97-12.58) |  | 26 (12.04) | 13.94 (7.18-27.06) |
| P80 (n=286) | 31 (10.84) | 2.91 (1.84-4.62) |  | 50 (17.48) | 7.33 (4.67-11.49) |  | 34 (11.89) | 24.26 (10.99-53.55) |
| **Elevated BP** |  |  |  |  |  |  |  |  |
| Recommended^*^ (n=215) | 19 (8.84) | 1.48 (0.87-2.52) |  | 21 (9.77) | 1.72 (1.03-2.89) |  | 7 (3.26) | 1.16 (0.51-2.67) |
| P90 (n=234) | 26 (11.11) | 2.18 (1.35-3.52) |  | 23 (9.83) | 1.87 (1.13-3.10) |  | 11 (4.70) | 1.98 (0.97-4.03) |
| P85 (n=341) | 33 (9.68) | 1.80 (1.15-2.80) |  | 29 (8.50) | 1.56 (0.98-2.48) |  | 15 (4.40) | 1.87 (0.98-3.58) |
| P80 (n=446) | 38 (8.52) | 1.48 (0.96-2.26) |  | 35 (7.85) | 1.42 (0.91-2.21) |  | 18 (4.04) | 1.73 (0.92-3.23) |
| **High FBG** |  |  |  |  |  |  |  |  |
| Recommended^*^ (n=88) | 8 (9.09) | 1.51 (0.69-3.31) |  | 8 (9.09) | 1.82 (0.83-4.00) |  | 7 (7.95) | 3.33 (1.38-8.06) |
| P90 (n=148) | 12 (8.11) | 1.26 (0.66-2.37) |  | 11 (7.43) | 1.29 (0.67-2.51) |  | 8 (5.41) | 2.13 (0.96-4.74) |
| P85 (n=217) | 19 (8.76) | 1.38 (0.81-2.35) |  | 14 (6.45) | 1.10 (0.61-2.00) |  | 11 (5.07) | 2.04 (1.00-4.17) |
| P80 (n=288) | 26 (9.03) | 1.49 (0.93-2.40) |  | 19 (6.60) | 1.16 (0.68-1.97) |  | 15 (5.21) | 2.33 (1.21-4.47) |
| **High TG** |  |  |  |  |  |  |  |  |
| Recommended^*^ (n=64) | 6 (9.38) | 1.95 (0.79-4.80) |  | 13 (20.31) | 5.92 (2.92-11.98) |  | 6 (9.38) | 4.82 (1.86-12.52) |
| P90 (n=150) | 14 (9.33) | 1.73 (0.95-3.16) |  | 21 (14.00) | 3.09 (1.82-5.27) |  | 11 (7.33) | 3.62 (1.76-7.45) |
| P85 (n=220) | 21 (9.55) | 1.80 (1.08-3.01) |  | 30 (13.64) | 3.25 (2.02-5.21) |  | 12 (5.45) | 2.62 (1.31-5.24) |
| P80 (n=291) | 24 (8.25) | 1.50 (0.92-2.44) |  | 37 (12.71) | 3.15 (2.01-4.92) |  | 17 (5.84) | 3.06 (1.62-5.79) |
| **Low HDL-C** |  |  |  |  |  |  |  |  |
| Recommended^*^ (n=76) | 9 (11.84) | 2.36 (1.12-4.97) |  | 9 (11.84) | 2.58 (1.21-5.47) |  | 7 (9.21) | 4.58 (1.92-10.92) |
| P10 (n=132) | 10 (7.58) | 1.38 (0.69-2.75) |  | 18 (13.64) | 2.90 (1.65-5.08) |  | 9 (6.82) | 3.11 (1.43-6.74) |
| P15 (n=200) | 15 (7.50) | 1.26 (0.71-2.25) |  | 21 (10.50) | 2.04 (1.21-3.44) |  | 10 (5.00) | 2.14 (1.03-4.46) |
| P20 (n=277) | 23 (8.30) | 1.42 (0.87-2.32) |  | 26 (9.39) | 1.83 (1.13-2.95) |  | 14(5.05) | 2.26 (1.17-4.39) |

Multi-class logistic regression models were performed separately for each cardiovascular risk factor adjusting for sex, age, sleep duration, screen time, physical activity, and intake of vegetables and fruits.

^*^Recommended guideline from the Society of Pediatrics, Chinese Medical Association.

P90, P85 and P80 represented the corresponding sex- and age-specific percentile values of waist circumference, systolic/diastolic BP, FBG and TG based on the present population, and P10, P15 and P20 for HDL-C.

†Percentages are calculated as: (the number of subjects with CR or EH or CH) / (the number of those with specific CV risk factors) ×100%.

BP, blood pressure; CH, concentric hypertrophy; CI, confidence interval; CR, concentric remodeling; CV, cardiovascular; EH, eccentric hypertrophy; FBG, fasting blood glucose; HDL-C, high-density lipoprotein cholesterol; LVG, left ventricular geometric; OR, odds ratio; TG, triglycerides.
